# Has loneliness and poor resilient coping influenced the magnitude of psychological distress among apparently healthy Indian adults during the lockdown? Evidence from a rapid online nation-wide cross-sectional survey

**DOI:** 10.1371/journal.pone.0245509

**Published:** 2021-01-14

**Authors:** Arista Lahiri, Sweety Suman Jha, Rudraprasad Acharya, Abhijit Dey, Arup Chakraborty

**Affiliations:** 1 Dept. of Community Medicine, College of Medicine and Sagore Dutta Hospital, Kolkata, West Bengal, India; 2 Dept. of Preventive and Social Medicine, All India Institute of Hygiene and Public Health, Kolkata, West Bengal, India; 3 Dept. of Psychiatry, Medical College and Hospital, Kolkata, West Bengal, India; 4 WHO RNTCP Technical Support Network, West Bengal, India; 5 Dept. of Community Medicine, Medical College and Hospital, Kolkata, West Bengal, India; University of Western Australia, AUSTRALIA

## Abstract

**Background:**

The burden of psychological distress is increasing with the spread of the pandemic and also with the enforcement of its containment measures. The aim of this research was to determine the proportion of self-reported psychological distress, loneliness and degrees of resilient coping, and to also investigate the relationship of loneliness, coping and other variables with psychological distress among apparently healthy Indians during nation-wide lockdown period.

**Methods:**

A cross-sectional, region-stratified survey using pre-designed pre-tested Google form disseminated via different social media platforms was conducted. A total of 1249 responses were analysed all over India. The form enquired about Socio-demographic profile, awareness on COVID pandemic and cases in the surroundings. UCLA Loneliness scale, Brief resilience and coping scale (BRCS) and Psychological distress scale (K6) assessed self-reported loneliness, coping and psychological distress, respectively. Special regressor technique adjusting for endogeneity and heteroskedasticity was used to extract the average marginal effects.

**Results:**

Majority of the respondents were 18–35 years old, male, single and urban residents. News media, social media mostly acted as sources of information regarding COVID related news. Overall, 54.47% (95% CI: 51.39–57.53%) and 38.39% (95% CI: 35.57–41.29%) were reported to be lonely and had low resilient coping ability respectively. Around 44.68% had high risk of developing psychological distress. Being a student (average marginal effect coefficient (AME_Coef_)_._: -0.07, 95% CI: [-0.12, -0.01]) and perceiving lockdown as an effective measure (AME_Coef_: -0.11, 95% CI: [-0.19, -0.03]) were protective against psychological distress. Psychological distress was associated with male respondents (AME_Coef_ 0.07, 95% CI: [0.02, 0.11]), low or medium resilient copers (AME_Coef_ 0.89, 95% CI: [0.17, 1.61]), and perceiving a serious impact of social distancing measures (AME_Coef_ 0.17, 95% CI: [0.09, 0.26]).

**Conclusions:**

Psychological distress among Indian population during lockdown was prevalent. Poor coping ability and perceiving social distancing to have a serious impact was found to be significantly contributing to psychological distress. Appropriate measures to address these issues would be beneficial for the community mental health.

## Introduction

As the novel coronavirus (COVID-19 or n-CoV-2019 or SARS-Cov-2) pandemic sweeps across the world, causing a serious impediment to the general health of the population and economic growth, it is causing widespread concern, fear and stress resulting in a deranged psychological well-being, all of which are natural and normal reactions to the changing and uncertain situation that everyone finds themselves in [1, 2]. Several researchers in their studies indicated towards a high burden of psychological distress often associated with an adverse perceived severity associated with the spread of the pandemic and also the containment measures like lockdown, mandatory use of facemask, social distancing etc. [3–8]. Globally the responses at national levels often fronted with lock-down enforcement have been challenging to the residents [8]. The psychological aspects in this regard need to be studied synergistically focusing the COVID response and the adaptive response to lock-down, especially in Indian context since the burden of morbidity tend to get out of proportion very frequently [9, 10].

There are several factors that influence psychological distress which may largely be due to variable range of coping [11, 12]. In an Australian study conducted during the equine influenza in 2007, presence of infection in the immediate surrounding of one’s habitat played a key role behind psychological distress [13]. Biological variation of psychological distress with age and gender is also known [4, 13, 14]. Certain social factors always play a part, causally or non-causally, e.g. residence, status of employment, level of education, marital status and living arrangement etc [5, 15]. Undoubtedly the COVID-19 pandemic in India and the unprecedented endeavour to stop the spread of the disease through nation-wide strict lockdown implementation induced a certain level of stress and uncertainty among the individuals especially the adult population of the country [7]. It can be hypothesized that the individual’s perception of severity of the pandemic and the effect of lockdown measures will influence the level of distress [4, 7].

Poor mental health is linked to plethora of social disharmonious outcomes like domestic violence, abuse, school dropouts, child labour, gender discrimination or may be even geriatric negligence and abuse, which act in synergy to tilt the scales of psychosocial wellbeing to an unfavourable degree. Undeniably the burden of psychological ill-effect is important from a public health point-of-view, as with gradual resumption of the activities, i.e. the ‘unlocking’ of the nation, it is necessary to understand the mental state of the workforce of the nation and take remedial actions at the earliest through policy-decisions and implementations. With implementation of lockdown, and social distancing the role of digital media increased manifolds in terms of communication and source of information. It is justified rationally also from feasibility perspective to survey the apparently healthy adults through online data collection technique, that will by virtue of the design account for the baseline effect of variable use of digital media. The aim of the current study was to determine the proportion of self-reported psychological distress, loneliness and degrees of resilient coping among the respondents during nation-wide lockdown period. The study also investigated the relationship of loneliness, coping and other variables with psychological distress.

## Materials and methods

### Study type and population

An observational analytical online questionnaire-based survey with cross-sectional design was conducted among the social media users from India. The data collection for this study was conducted over one-month duration starting from April 17 –May 16, 2020. Clearance was obtained from the Institutional Ethics Committee of Medical College, Kolkata, West Bengal. Individuals who had access to social media platforms like Facebook^®^ or Twitter^®^ or Instagram^®^ or LinkedIn^®^, were considered as the study population. Also, access to WhatsApp^®^ was considered important from ease of communication perspective. Adult population (18–65 years) and Indian by nationality who were currently living in India since the beginning of the country-wide Lockdown on March 25, 2020, with clear understanding of English were included in the study. Those having critical illness or under palliative care were excluded based on their self-declaration. Participants diagnosed with any cognitive or psychiatric illness or those on psychotropic or sedative medication were also excluded from this study through skip patterns incorporated in the online questionnaire. Those who participated in the study provided an online written informed consent before responding to the online questionnaire.

### Selection of the participants

**Fig 1:**
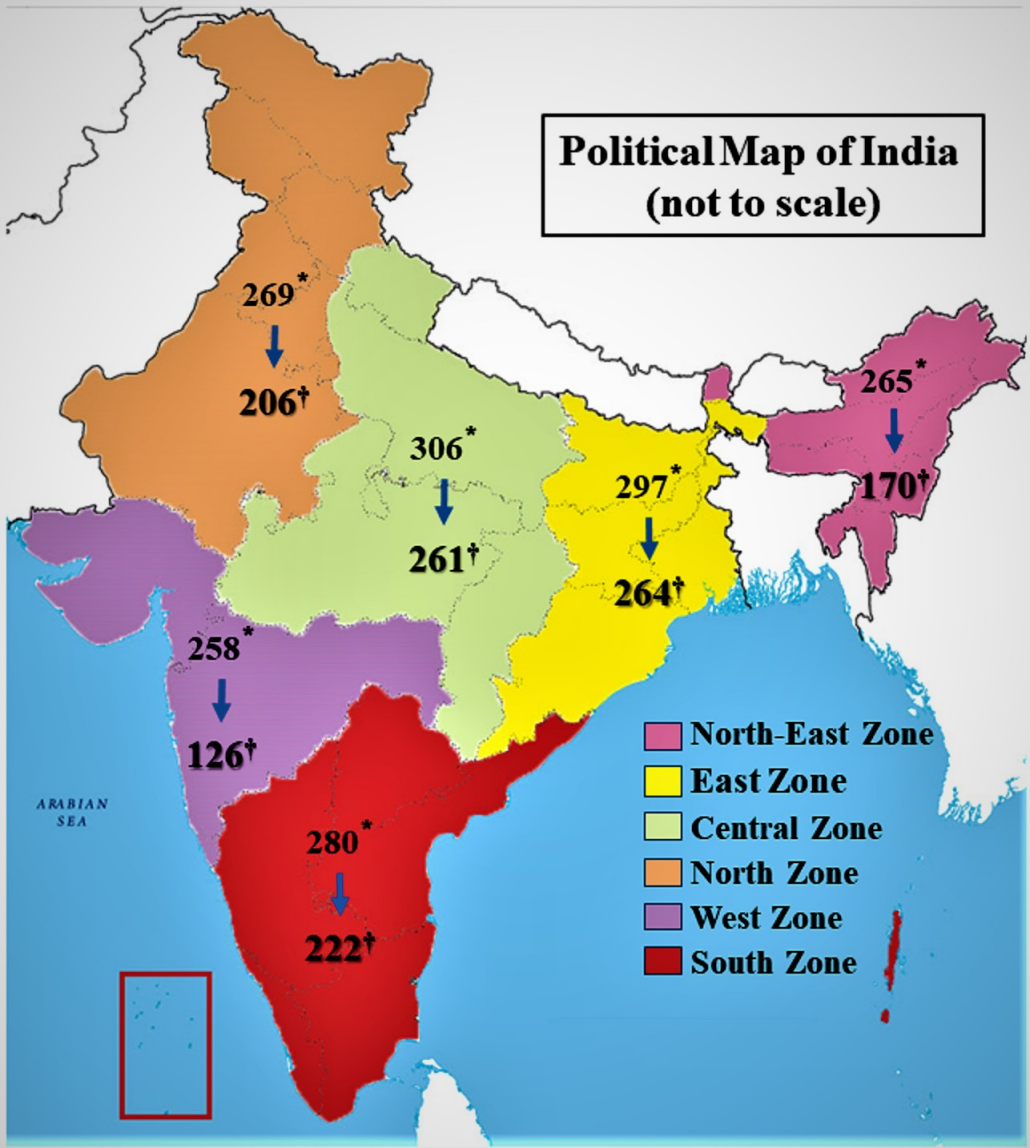
Sampling zones in India and participants selected from each zone. * indicates the number of participants in the respective zones (selected based on their digital profiles) who accessed the data collection form, ^†^ indicates who responded and submitted the form (includes those who moved from other zones and currently residing in a different zone, but not updated it their digital profile). For details of sample selection refer to S1 File.

Based on the observations from a pilot study (proportion of self-reported psychological distress ~ 40%) the minimum required sample size was estimated to be 1153, considering 5% precision and 90% power of the study with a design effect of 2. The response rate was taken as minimum 80% out of total distribution. This yielded a target sample size of ~ 1440, for which optimally around 240 responses were targeted from each zone. The zones for the study and number of respondents from each zone has been shown in Fig 1. Social media platforms were searched by name of different states and union-territories as per the zones, where zones were considered as strata for the sample. The resultant open-ended list was used to select desired number of participants in different zones through random number sequences. The participants were contacted through their available contact information (email or WhatsApp number) and the Google form was shared. Finally, a total of 1249 responses were included in the final analysis with 264 from Eastern zone, 206 from Northern zone, 126 from Western zone, 222 from Southern zone, 261 from Central zone and 170 from North-eastern zone. The strategy of zonal stratification and participant selection is outlined in the S1 File. The collected data were validated through re-test with the same questionnaire sent through email to randomly selected 10% respondents in each day (Refer S2 File).

### Measurements of different variables

The pre-designed pre-tested online questionnaire (Google form), used for data collection was comprised of questions on Socio-demographic and clinical information, awareness on COVID pandemic and cases in the surroundings, sources and levels of stress, levels of insomnia and anxiety. The questionnaire was provided in English language. The collected data using predesigned Google form (Google LLC, California, USA) was auto-entered into a linked generated Google sheet (Google LLC, California, USA). The questionnaire was designed through brain-storming sessions with subject experts from the disciplines of psychiatry, clinical psychology, and social sciences. The participants were enquired about their age, gender, residence, current living arrangement, employment status, whether going out to office/institution highest, educational level, and sources of information regarding COVID pandemic. Apart from those married, those who were not currently married, and not in any relationship were considered single, and those living-in with the partner or in a relationship were considered as ‘others’ in case of marital status. The respondents were also enquired whether there were any confirmed and/or suspect cases in their immediate social surrounding and/or relatives and close friends.

Loneliness was measured with the help of validated three-item UCLA Loneliness scale [16] and categorized into lonely and not lonely based on the total score obtained–total score of 6 and above indicating loneliness. Coping was measured by the brief resilience and coping scale (BRCS) [17], another validated tool which classified the participants into three categories based on the total scale score, viz. high resilient copers, medium resilient copers and low resilient copers. Psychological distress, considered the outcome variable was measured by Psychological distress scale (K6 scale) [18]. The item specific scores were added and based on the total scores, the participants were categorized into low risk and high risk for developing psychological distress. The Cronbach’s alpha values for UCLA Loneliness scale, BRCS and K6 scale were 0.81, 0.78 and 0.90 respectively in the current study sample. In consultation with mental health practitioners and clinical psychologists, three items on five-point Likert-type scale were included measuring the perception regarding effect of the pandemic, the effectiveness of lockdown and last but not the least the impact of imposed physical distancing measures separately.

### Statistical analysis

Statistical analysis was performed using STATA 14.2 software (StataCorp LLC, College Station, Texas, USA). Confidentiality was maintained while cleaning and storing the data for analysis. The proportions or prevalence of different factors were calculated weighing for migration-adjusted response proportions (Refer Table S2.2 in S2 File). For these proportions 95% confidence intervals (95% CIs) were obtained using clustered robust standard error taking into account the clustering effect of the zones. The categorical variables in the study were tested for trends across different zones by chi-square test. The differences in median values of the continuous study variables were tested by Kruskal-Wallis test. While analysing different factors for their association with anxiety levels, the regression models were built combining responses from all the zones. Partially complete responses i.e. those with missing values were dropped from the regression model.

While analysing the relationship between different factors and the different levels of self-reported psychological distress, self-reported loneliness and resilient coping were endogenous to the analytical model. Special regressor technique [19, 20] was implemented to understand this multi-variable relationship. In order to obtain a better statistical fit resilient coping was considered in two categories, viz. high resilient copers and low or medium resilient copers. Work/study from home and being married were used as instruments for the endogenous variables respectively. Considering the Likert-type responses to the questions of perceptions regarding effect of pandemic, effectiveness of lockdown, and impact of physical distancing to be continuous, perception regarding impact of physical distancing was taken as the special regressor (kurtosis: 2.614) in the final model, while the other two perception variables considered exogenous explanatory variables. The model was adjusted for the effects of age, sex, area of residence, education, employment status and the knowledge about any confirmed and/or suspected cases of COVID19 in the surrounding. Variables describing sources of information (healthcare worker, social media, and traditional news media) were applied to control for probable heteroskedasticity. Average marginal effect coefficients (AME_Coef._) of the explanatory variables were obtained from average index function (AIF) with kernel density estimator [21]. Bootstrap standard errors (default seed with ten bootstrap replications) were calculated to obtain 95% CI of the estimates. The overall model was considered statistically fit as the instrumental variables were statistically significant in instrumenting the endogenous predictors (P_χ2_ = 0.0022), and the combination of exogenous variables in the model was also statistically significant (P <0.001).

## Results

### Background information

In the final analysis 1249 responses were included. While 21.14% responses were from Eastern zone, Western zone contributed 10.09% responses. The background information of the respondents is summarized in Table 1. The median age of the respondents was 28 years with an inter-quartile range (IQR) of 14 years. Overall, 71.19% (95% CI: 62.73–78.39%) respondents belonged to younger age group (18–35 years) with 4.03% (95% CI: 2.97–5.44%) belonging to 51–65 years age group. The overall proportion of those currently married was 42.13% (95% CI: 33.91–50.82%). Majority respondents were male and from urban areas. About 13.45% (95% CI: 11.14–15.83%) were unemployed at the time of this survey. It was noted that overall, 20.63% (95% CI: 18.75–22.66%) were going out to attend workplace regularly. Among the respondents 11.86% (95% CI: 11.48–12.24%) were living alone, while among those currently married, 3.97% were living alone. However, among those not currently living alone, 63.93% were living with their parents/ parents-in-laws and 6.12% were living with grandparents/ grandparents-in-law. Almost all the participants (96.38%, 95% CI: 93.71–97.95%) cited news media as a source of information regarding the disease. More than half of the respondents were receiving information from the social media, while 36.79% (95% CI: 34.35–39.30%) reported to have received information about COVID19 from healthcare worker(s).

**Table 1:** Socio-demographic profile of the respondents.

Socio-demographic factors	Zones						Total	P-value, χ^2^, df
	East	North	West	South	Central	North-East		
**Age group**	**n = 264**	**n = 206**	**n = 126**	**n = 222**	**n = 261**	**n = 170**	**n = 1249**	
***18–35 years***	195 (73.86)	169 (82.04)	76 (60.32)	135 (60.81)	184 (70.50)	126 (74.12)	885 (70.86)	0.000, 65.33, 20
***36–50 years***	58 (21.97)	32 (15.53)	45 (35.71)	74 (33.33)	67 (25.67)	38 (22.35)	314 (25.14)	
***51–65 years***	11 (4.17)	5 (2.43)	5 (3.97)	13 (5.86)	10 (3.83)	6 (3.53)	50 (4.00)	
**Gender**	**n = 264**	**n = 206**	**n = 126**	**n = 222**	**n = 261**	**n = 170**	**n = 1249**	
***Female***	125 (47.35)	93 (45.15)	49 (38.89)	89 (40.09)	108 (41.38)	83 (48.82)	547 (43.80)	0.274, 6.34, 5
***Male***	139 (52.65)	113 (54.85)	77 (61.11)	133 (59.91)	153 (58.62)	87 (51.18)	702 (56.20)	
**Marital Status**	**n = 264**	**n = 206**	**n = 126**	**n = 222**	**n = 261**	**n = 170**	**n = 1249**	
***Single***	154 (58.33)	136 (66.02)	53 (42.06)	99 (44.59)	151 (57.85)	109 (64.12)	702 (56.20)	0.000, 41.69, 10
***Married***	104 (39.39)	67 (32.52)	73 (57.94)	119 (53.60)	108 (41.38)	58 (34.12)	529 (42.35)	
***Other***	6 (2.27)	3 (1.46)	0 (0.00)	4 (1.80)	2 (0.77)	3 (1.76)	18 (1.44)	
**Residence**	**n = 264**	**n = 206**	**n = 126**	**n = 222**	**n = 261**	**n = 170**	**n = 1249**	
***Rural***	46 (17.42)	36 (17.48)	16 (12.70)	36 (16.22)	50 (19.16)	35 (20.59)	219 (17.53)	0.567, 3.88, 5
***Urban***	218 (82.58)	170 (82.52)	110 (87.30)	186 (83.78)	211 (80.84)	135 (79.41)	1030 (82.47)	
**Highest Educational Level**	**n = 263**	**n = 204**	**n = 126**	**n = 221**	**n = 259**	**n = 170**	**n = 1243**	
***Up to completed Class XII***	29 (11.03)	27 (13.24)	12 (9.52)	23 (10.41)	35 (13.51)	27 (15.88)	153 (12.31)	0.316, 11.55, 10
***Non-Professional*** ***(Graduates and above)***	109 (41.44)	89 (43.62)	47 (37.30)	79 (35.75)	91 (35.14)	57 (33.53)	472 (37.97)	
***Professional***	125 (47.53)	88 (43.14)	67 (53.17)	119 (53.85)	133 (51.35)	86 (50.59)	618 (49.72)	
**Occupation**	**n = 264**	**n = 206**	**n = 126**	**n = 222**	**n = 261**	**n = 170**	**n = 1249**	
***Unemployed***	36 (13.64)	22 (10.68)	18 (14.29)	35 (15.77)	38 (14.56)	17 (10.00)	166 (13.29)	0.000, 35.91, 10
***Employed or Home-maker***	157 (59.47)	111 (53.88)	88 (69.84)	146 (65.77)	157 (60.15)	88 (50.76)	747 (59.81)	
***Student***	71 (26.89)	73 (35.44)	20 (15.87)	41 (18.47)	66 (25.29)	65 (38.24)	336 (26.90)	
**Living arrangement**	**n = 264**	**n = 206**	**n = 126**	**n = 222**	**n = 261**	**n = 170**	**n = 1249**	
***Living alone***	32 (12.12)	25 (12.14)	15 (11.90)	25 (11.26)	31 (11.88)	20 (11.76)	148 (11.85)	1.000, 0.11, 5
***Living with Parents/*** ***Parents In-law***	155 (58.71)	131 (63.59)	58 (46.03)	109 (49.10)	146 (55.94)	107 (62.94)	706 (56.53)	0.003, 18.21, 5
***Living with Grandparents/ Grandparents In-law***	14 (5.30)	9 (4.37)	11 (8.73)	12 (5.41)	16 (6.13)	5 (2.94)	67 (5.36)	0.360, 5.48, 5
***Living with Spouse/Partner***	85 (32.20)	58 (28.16)	58 (46.03)	95 (42.79)	89 (34.10)	53 (31.18)	438 (35.07)	0.002, 18.99, 5
***Living with Children***	53 (20.08)	28 (13.59)	42 (33.33)	65 (29.28)	54 (20.69)	24 (14.12)	266 (21.30)	0.000, 32.14, 5
**Sources of Information regarding COVID related news**	**n = 264**	**n = 206**	**n = 126**	**n = 222**	**n = 261**	**n = 170**	**n = 1249**	
***Healthcare Worker***	101 (38.26)	80 (38.83)	37 (29.37)	75 (33.78)	97 (37.16)	66 (38.82)	456 (36.50)	0.446, 4.76, 5
***Social media***	133 (50.38)	110 (53.40)	67 (53.17)	116 (52.25)	127 (48.66)	88 (51.76)	641 (51.32)	0.918, 1.45, 5
***News media***	257 (97.35)	203 (98.54)	119 (94.44)	212 (95.50)	246 (94.25)	167 (98.24)	1204 (96.40)	0.064, 10.43, 5
***Other sources***	153 (57.95)	128 (62.14)	66 (52.38)	129 (58.11)	149 (57.09)	97 (57.06)	722 (57.80)	0.668, 3.21, 5

Numbers in the parentheses represent percentage values of the response categories within each zone/overall. χ^2^: Chi-square value for the test of trend within different zones, df: degrees of freedom.

### Information about cases, and perceptions about pandemic, lockdown and social distancing

At the time of the survey, majority of the respondents were confident that there was no confirmed or suspected case of COVID19 in their social surroundings or among their families and close friends. The proportion of those having any confirm and/or suspect case in their surrounding and/or families and friends was 17.83% (95% CI: 14.73–21.42%). The information about cases are summarized in Table 2.

**Table 2:** Knowledge about confirmed or suspected cases of COVID19 in social surrounding or amongst family/friends/relatives.

Knowledge about COVID19 cases	Zones						Total	P-value, χ^2^, df
	East	North	West	South	Central	North-East		
**Confirmed case in immediate social surrounding**	**n = 264**	**n = 206**	**n = 122**	**n = 218**	**n = 255**	**n = 169**	**n = 1234**	
***Present***	24 (9.09)	21 (10.19)	8 (6.56)	15 (6.88)	20 (7.84)	13 (7.69)	101 (8.18)	0.810, 6.06, 10
***Absent***	205 (77.65)	161 (78.16)	93 (76.23)	167 (76.61)	192 (75.29)	133 (78.70)	951 (77.07)	
***Don’t know***	35 (13.26)	24 (11.65)	21 (17.21)	36 (16.51)	43 (16.86)	23 (13.61)	182 (14.75)	
**Suspected case in immediate social surrounding**	**n = 264**	**n = 206**	**n = 122**	**n = 218**	**n = 255**	**n = 169**	**n = 1234**	
***Present***	35 (13.26)	29 (14.08)	11 (9.02)	20 (9.17)	26 (10.20)	22 (13.02)	143 (11.59)	0.226, 12.96, 10
***Absent***	152 (57.58)	120 (58.25)	67 (54.92)	116 (53.21)	130 (50.98)	92 (54.44)	677 (54.86)	
***Don’t know***	77 (29.17)	57 (27.67)	44 (36.07)	82 (37.61)	99 (38.82)	55 (32.54)	414 (33.55)	
**Confirmed case within your relatives/ friends**	**n = 264**	**n = 206**	**n = 122**	**n = 218**	**n = 255**	**n = 169**	**n = 1234**	
***Present***	17 (6.44)	12 (5.83)	6 (4.92)	7 (3.21)	9 (3.53)	6 (3.55)	57 (4.62)	0.831, 5.80, 10
***Absent***	225 (85.23)	176 (85.44)	104 (85.25)	189 (86.70)	222 (87.06)	144 (85.21)	1060 (85.90)	
***Don’t know***	22 (8.33)	18 (8.74)	12 (9.84)	22 (10.09)	24 (9.41)	19 (11.24)	117 (9.48)	
**Suspected case within your relatives/ friends**	**n = 264**	**n = 206**	**n = 122**	**n = 218**	**n = 255**	**n = 169**	**n = 1234**	
***Present***	11 (4.17)	7 (3.40)	4 (3.28)	5 (2.29)	8 (3.14)	4 (2.37)	39 (3.16)	0.387, 10.63, 10
***Absent***	209 (79.17)	159 (77.18)	104 (85.25)	184 (84.40)	210 (82.35)	129 (76.33)	995 (80.63)	
***Don’t know***	44 (16.67)	40 (19.42)	14 (11.48)	29 (13.30)	37 (14.51)	36 (21.30)	200 (16.21)	

Numbers in the parentheses represent percentage values of the response categories within each zone/overall. χ^2^: Chi-square value for the test of trend within different zones, df: degrees of freedom.

The perception of the respondents about the effect of the pandemic, lockdown measures and the strict physical distancing regulations on their daily lives, is represented in Table 3. The proportion of participants who perceived the effect of pandemic to be very sever, lockdown to be very much effective and physical distancing to have very serious effect were 19.23% (95% CI: 17.71–20.85%), 16.67% (95% CI: 14.61–18.97%) and 15.66% (14.03–17.44%) respectively. On the other hand, 3.58% (95% CI: 3.01–4.26%), 1.81% (95% CI: 1.02–3.20%), 6.28% (95% CI: 4.74–8.28%) respectively, perceived no effects.

**Table 3:** Perception about the pandemic, lockdown and social distancing measures on daily life.

Perception on	Zones						Total	P-value, χ^2^, df
	East	North	West	South	Central	North-East		
**Pandemic**	**n = 264**	**n = 206**	**n = 122**	**n = 221**	**n = 260**	**n = 170**	**n = 1243**	
***No effect***	10 (3.79)	7 (3.40)	1 (0.82)	9 (4.07)	9 (3.46)	8 (4.71)	44 (3.54)	0.575, 18.19, 20
***Minimum effect***	27 (10.23)	26 (12.62)	18 (14.75)	24 (10.86)	20 (7.69)	27 (15.88)	142 (11.42)	
***Somewhat***	105 (39.77)	76 (36.89)	56 (45.90)	86 (38.91)	110 (42.31)	67 (39.41)	500 (40.23)	
***Severe effect***	70 (26.52)	56 (27.18)	31 (25.41)	60 (27.15)	68 (26.15)	35 (20.59)	320 (25.74)	
***Very severe effect***	52 (19.70)	41 (19.90)	16 (13.11)	42 (19.00)	53 (20.38)	33 (19.41)	237 (19.07)	
**Lockdown**	**n = 264**	**n = 206**	**n = 122**	**n = 221**	**n = 260**	**n = 170**	**n = 1243**	
***Not effective***	6 (2.27)	1 (0.49)	5 (4.10)	6 (2.71)	6 (2.31)	0 (0.00)	24 (1.93)	0.407, 20.83, 20
***Minimally effective***	22 (8.33)	18 (8.74)	15 (12.30)	21 (9.50)	21 (8.08)	19 (11.18)	116 (9.33)	
***Somewhat effective***	100 (37.88)	82 (39.81)	51 (41.80)	77 (34.84)	93 (35.77)	60 (35.29)	463 (37.25)	
***Effective***	93 (35.23)	70 (33.98)	41 (33.61)	82 (37.10)	93 (35.77)	58 (34.12)	437 (35.16)	
***Very much effective***	43 (16.29)	35 (16.99)	10 (8.20)	35 (15.84)	46 (18.08)	33 (19.41)	203 (16.33)	
**Social Distancing**	**n = 264**	**n = 206**	**n = 122**	**n = 221**	**n = 260**	**n = 170**	**n = 1243**	
***No impact***	14 (5.30)	10 (4.85)	15 (12.30)	13 (5.88)	15 (5.77)	15 (8.82)	82 (6.60)	0.608, 17.69, 20
***Minimum impact***	47 (17.80)	35 (16.99)	26 (21.31)	37 (16.74)	40 (15.38)	28 (16.47)	213 (17.14)	
***Somewhat impact***	101 (38.26)	82 (39.81)	39 (31.97)	83 (37.56)	99 (38.08)	68 (40.00)	472 (37.97)	
***Serious impact***	62 (23.48)	49 (23.79)	30 (24.59)	50 (22.62)	63 (24.23)	32 (18.82)	286 (23.01)	
***Very serious impact***	40 (15.15)	30 (14.56)	12 (9.84)	38 (17.19)	43 (16.54)	27 (15.88)	190 (15.29)	

Numbers in the parentheses represent percentage values of the response categories within each zone/overall. χ^2^: Chi-square value for the test of trend within different zones, df: degrees of freedom.

### Loneliness, coping and psychological distress

The median scores for loneliness, resilient coping and psychological distress were respectively, 6 (IQR: 3), 14 (IQR: 4) and 10 (IQR:9). The zone-wise distribution of self-reported loneliness, resilient coping and psychological distress is depicted in Table 4. The zone-wise differences were not statistically significant. However, respondents from the North-Eastern zone reported highest proportion of loneliness (60.95%). More than 40% respondents from Eastern and Central zones were low resilient copers. Overall, 54.47% (95% CI: 51.39–57.53%) and 38.38% (95% CI: 35.57–41.29%) were reported to be lonely and had low resilient coping ability respectively. Around 45.24% (95% CI: 41.54–48.99%) were high risk of developing psychological distress.

**Table 4:** Loneliness and resilient coping and psychological distress among the respondents.

	Zones						Total	P-value, χ^2^, df
	East	North	West	South	Central	North-East		
**Loneliness**	**n = 264**	**n = 203**	**n = 122**	**n = 220**	**n = 257**	**n = 169**	**n = 1235**	
***Lonely***	142 (53.79)	110 (54.19)	66 (54.10)	122 (55.45)	133 (51.75)	103 (60.95)	676 (54.74)	0.587, 3.74, 5
***Not lonely***	122 (46.21)	93 (45.21)	56 (45.90)	98 (44.55)	124 (48.25)	66 (39.05)	559 (45.26)	
**Degree of Resilient Coping**	**n = 264**	**n = 206**	**n = 125**	**n = 220**	**n = 258**	**n = 169**	**n = 1242**	
***High resilient copers***	57 (21.59)	45 (21.84)	29 (23.20)	44 (20.00)	62 (24.03)	35 (20.71)	272 (21.90)	0.755, 6.68, 10
***Medium resilient copers***	101 (38.26)	89 (43.20)	55 (44.00)	90 (40.91)	91 (35.27)	73 (43.20)	499 (40.18)	
***Low resilient copers***	106 (40.15)	72 (34.95)	41 (32.80)	86 (39.09)	105 (40.70)	61 (36.09)	471 (37.92)	
**Psychological distress**	**n = 264**	**n = 205**	**n = 123**	**n = 219**	**n = 259**	**n = 169**	**n = 1239**	
***Low risk***	141 (53.41)	123 (60.00)	72 (58.54)	116 (52.97)	133 (51.35)	100 (59.17)	685 (55.29)	0.319, 5.87, 20
***High risk***	123 (46.59)	82 (40.00)	51 (41.46)	103 (47.03)	126 (48.65)	69 (40.83)	554 (44.71)	

Numbers in the parentheses represent percentage values of the response categories within each zone/overall. χ^2^: Chi-square value for the test of trend within different zones, df: degrees of freedom.

### Factors associated with psychological distress

The special regressor regression model adjusted for endogeneity, showing average marginal effects of the different factors associated with high risk for psychological distress are depicted in Table 5. Those having a lower degree of coping were at risk of psychological distress (AME_Coef._: 0.89, 95% CI: [0.17, 1.61]). A better perception regarding effectiveness of lockdown measures were found to be protective of psychological distress, however those perceiving a more severe impact of physical distancing measures were at risk for psychological distress. Both the relationships were statistically significant. Male respondents were at higher risk of psychological distress compared to females (AME_Coef._: 0.07, 95% CI: [0.02, 0.11]). Students were found to be protected from psychological distress (AME_Coef._: -0.07, 95% CI: [-0.12, -0.01]).

**Table 5:** Factors associated with psychological distress among the respondents.

Factors behind Psychological Distress	AME_Coef._ (95% CI)	P-value
Age*	0.02 (-0.04, 0.08)	0.489
**Gender** *(Ref*.: *Female)*		
*Male*	0.07 (0.02, 0.11)	0.007
**Area of residence** *(Ref*.: *Rural)*		
*Urban*	-0.03 (-0.10, 0.03)	0.348
**Education**		
*Graduate and above (Professional courses)* ^*†*^	0.00 (-0.53, 0.53)	0.996
*Graduate and above (Non-professional courses)* ^*†*^	0.03 (-0.48, 0.54)	0.908
*Below graduation*^*†*^	0.04 (-0.53, 0.61)	0.891
**Employment status**		
*Unemployed*^*†*^	-0.05 (-0.11, 0.02)	0.179
*Student*^*†*^	-0.07 (-0.12, -0.01)	0.013
**Living arrangement**		
*Living alone*^*†*^	0.00 (-0.12, 0.12)	0.995
*Living with parents*^*†*^	0.02 (-0.06, 0.11)	0.591
*Living with grandparents*^*†*^	0.13 (0.04, 0.22)	0.004
*Living with spouse*^*†*^	-0.02 (-0.12, 0.08)	0.673
*Living with children*^*†*^	0.01 (-0.04, 0.05)	0.698
**Self-reported loneliness**^**‡**^ *(Ref*.: *Not lonely)*		
*Lonely*	0.16 (-0.48, 0.80)	0.624
**Self-reported resilient coping ability**^**‡**^ *(Ref*.: *High resilient copers)*		
*Low or medium resilient copers*	0.89 (0.17, 1.61)	0.016
**Any confirmed &/or suspect case(s) in the surrounding** *(Ref*.: *Absent)*		
*Present*	0.01 (-0.05, 0.08)	0.671
**Perceptions regarding,**		
Effect of pandemic*	0.01 (-0.03, 0.04)	0.770
Effectiveness of lockdown*	-0.11 (-0.19, -0.03)	0.010
Impact of social distancing*^,^^#^	0.17 (0.09, 0.26)	0.000

Average marginal effect of the variables calculated based on average index function from the special regressor regression technique using Kernel density estimator. Special regressor model was statistically fit with valid instruments (P_χ2_ = 0.0022 for instrumental variables regression). AME_Coef._: Average marginal effects coefficient, CI: Confidence interval, Ref.: Reference category.

* Variables considered continuous in the model.

†Indicator variables with reference category being complement of the reported category

^‡^ Endogenous variables

^#^Special regressor.

## Discussion

### Key findings

Predominantly, the respondents were of younger age group, consistent with expertise of the younger segment in engaging in social media and use of smartphones. In consonance, social media was reported as the dominant source of information on the COVID situation. The proportion of male and female participants were comparable. While the proportion of currently single participants was higher than the ‘other’ category, may be owing to social desirability as per prevalent social norms. Majority of the participants perceived the effect of the pandemic, effectiveness of lockdown and impact of physical distancing on a higher scale, an expected response to a new and unaccustomed situation. Self-reported loneliness and poor coping was present among more than half of the participants, with a sizeable number of respondents being psychologically distressed. Poor coping ability, more serious perceived impact of the physical distancing measures, male gender, and currently living with senior members of the family (e.g. grandparents/grandparents-in-law) were associated with higher degrees of psychological distress. However, self-reported loneliness was not found to be statistically associated with psychological distress. Students, and those who considered lockdown to be an effective measure had a significant negative association with self-reported psychological distress.

### What is already known and what this study adds

Studies on psychological distress in India have mainly focused on the university and college students [22–24]. But objective assessment of psychological distress among adults especially in the context of pandemic has not been attempted by many researches, leaving avoid the current article attempts to fill in. On the other hand a recent article explored gaming as a means of coping focusing the college students [25], but evidence regarding the levels of coping and their prevalence is lacking in India. However, in an online survey among mostly the residents of Hubei province, China average proportion of mental well-being was 49.4%, while in Germany 50% respondents reported anxiety and psychological distress [14, 26]. In another online survey contemporary to the current study, mostly negative approach was reported during the later phase of lockdown [27]. Consistent with these observations, on overall calculation nearly 45% respondents were found to be at high risk of developing psychological distress, while more than half of the participants reported themselves as lonely and did not have high resilient coping ability. Similarly, Verma and Mishra (2020) reported 25%, 28% and 11.6% of the participants to be moderate to extremely severely depressed, anxious and stressed, respectively [28].

The respondents from the states of central zone were highest in proportion reporting psychological distress. This can be synchronized with increasing case burden in these states at the time of the study. Such stern inferencing, though conceptually pertinent, but requires ecological level of analysis, which has been not done in this study. Losada-Baltar et al. (2020) emphasized on the importance of a wider support for psychological well-being [29]. However, psychological distress was observed more among those who were living with senior members of their families (e.g. grandparents/ grandparent-in-laws) at the time of the study. Though somewhat in contrast to the Spanish study, researchers in Germany however impressed the fact that the psycho-social issues were more pertinent in terms of psychological turbulence like anxiety as compared to the experience with the disease itself [5, 26].

Interestingly age was not associated with psychological distress in the study sample. On the other hand, Ahmed et al. (2020) inferred in their study and pointed out that 21–40 years age group was psychologically vulnerable [14]. They also reported that employment of the respondents did not play any role [14]. Verma and Mishra (2020) on the other hand reported employment to be an important correlate of depressive and anxiety symptoms [28]. In the current study sample unemployment was not observed to be statistically associated with psychological distress, but students were found protected. Flexibility to adopt to changes owing to pandemic and its control measures, in the midst of a prevalent ‘infodemic’ among the students can be a plausible explanation.

Those who perceived more serious impact of imposed physical distancing measures, were found to be more at risk of psychological distress. However, those perceiving lockdown to be effective were noted to be protected, which conceptually consistent. Those who were medium or low resilient copers had the strongest association with psychological distress among the factors studied in the model in this study. These were in synchrony with the conceptual framework and prevalent knowledge [26, 29, 30]. But the current study did not find any statistically significant relationship between loneliness and psychological distress.

Rehman et al. (2020) in their study during early phases of lockdown in India found that there was no gender difference in terms of depression, anxiety and stress [7]. In the present analysis male respondents were found at risk contrasting the findings from a Spanish study, but was in consonance with the findings by Verma and Mishra (2020) [28, 29]. Overall the psychological ill-effect as evident from the current study supported the general notion that lockdown imposed in various countries to contain the spread of the COVID-19 is associated with various psychosocial problems [8].

### Strengths and limitations

The current study is probably the first online survey in India to utilize a probability sampling technique in its design. Though the minimum required sample size was reached, but responses from several zones were suboptimal. However, by virtue of response weights and cluster adjusted robust standard errors the prevalence measures were considered valid based on the sample. The current study utilized the special regressor model to explain binary dependent variable in light of binary endogenous explanatory variables. This adds to the robustness of the study. While use of a discrete-choice response variable as special regressors may be argued against, but it was within the theoretical and conceptual bounds of the implemented model. With even a higher power, path analysis with structural equations modeling might have been a better alternative.

Considering the distribution of responses over the duration of data collection, majority of the responses were obtained during the early phases with a decreasing trend in new responses since the later second week of data collection period. Though it is a known fact that addiction is an important correlate of mental health, but enquiry on the same through self-reported responses were not considered in the final survey [8, 28]. High non-response rate to these questions and probable socially desirable response during the pre-testing phase led to exclusion of the addiction related items in the final survey. For the same reason, issues like job loss, economic insecurity, domestic violence, abuse etc. could not be explored in the context of psychological distress. Self-reported responses are often considered biased to some extent, but response validation in this study added a strength in terms of data integrity and validity.

## Conclusions

The burden of psychological distress among Indian population during the later phases of the lock-down cannot be undermined. The important modifiable factors behind psychological distress were poor coping, and more serious perception about physical distancing measures. Control of infection is in fact the best strategy to reduce the burden of distress, as it will not only reduce the number of cases, but will decrease the associated psychological distress also. Intensive awareness activities focusing on proper knowledge on the magnitude of the pandemic, mitigating rumors and also addressing psychosocial concerns are a necessity. Strategies focusing on mass psychological counselling to boost the coping ability, improved social connectivity through digital group activity maintaining distancing are required. Last but not the least, with progress of unlock phase, the health system must be made equipped to handle the dual burden of COVID19 infection and the piling psychological distress in the communities.

## Supporting information

S1 File Selection of participants according to composition of zonal strata. (DOCX)

S2 File Validation sample and result of test-retest reliability. (DOCX)
