# Supplementary material for: Has loneliness and poor resilient coping influenced the magnitude of psychological distress among apparently healthy Indian adults during the lockdown? Evidence from a rapid online nation-wide cross-sectional survey
Source: PLoS One. 2021 Jan 14;16(1):e0245509. doi: 10.1371/journal.pone.0245509 (PMC7808626; doi:10.1371/journal.pone.0245509)
Supplement: S1 File — (DOCX) [file pone.0245509.s001.docx]

**Selection of participants according to composition of zonal strata:**

**Table S1.1. Composition of the zonal strata in terms of different states of India.**

| Zone  (Sampling Strata) | States |
| --- | --- |
| East Zone | Bihar, Jharkhand, Odisha, and West Bengal |
| North Zone | Chandigarh, Delhi, Haryana, Himachal Pradesh, Jammu and Kashmir, Ladakh, Punjab, and Rajasthan |
| West Zone | Dadra and Nagar Haveli and Daman and Diu, Goa, Gujarat, and Maharashtra |
| South Zone | Andhra Pradesh, Karnataka, Kerala, Puducherry, Tamil Nadu, Telangana, Andaman and Nicobar Islands, Lakshadweep. |
| Central Zone | Chhattisgarh, Madhya Pradesh, Uttarakhand and Uttar Pradesh |
| North-East Zone | Sikkim, Assam, Arunachal Pradesh, Manipur, Meghalaya, Mizoram, Nagaland and Tripura |

**Zone-wise distribution of response numbers:**

**Table S1.2. Zone-wise distribution of response dynamics among the participants.**

| Strata | Primary distribution *^a^* | Added (Secondary) distribution *^b^* | Number of participants approached based on sampling protocol *^c^* | Number of participants responded (based on sampling zone) *^d^* | Response rate *^e^* | Responses obtained as per zonal migration (based on current residence) *^f^* | Migration proportion among respondents *^g^* |
| --- | --- | --- | --- | --- | --- | --- | --- |
| East zone | 240 | 57 | 297 | 215 | 0.72 | 264 | 0.23 |
| North Zone | 240 | 29 | 269 | 210 | 0.78 | 206 | - 0.02 |
| West Zone | 239 | 19 | 258 | 201 | 0.78 | 126 | - 0.37 |
| South Zone | 235 | 55 | 280 | 207 | 0.74 | 222 | 0.07 |
| Central Zone | 240 | 66 | 306 | 217 | 0.71 | 261 | 0.20 |
| North-East Zone | 237 | 28 | 265 | 199 | 0.75 | 170 | - 0.14 |

***^a^*** Participants who were provided with the google form based on sampling strategy.

***^b^*** Further distribution was done to a second set of participants when response from the primary participant was not obtained within three days of questionnaire delivery.

***^c^*** Number of participants approached based on sampling protocol = {Primary distribution + Added (Secondary) distribution}

***^d^*** Shows the number of participants who responded, based on their zone as per their digital profile (i.e. as per the sampling strategy)

***^e^*** Response rate was calculated as, {Number of participants responded (based on sampling zone) $\div$ Number of participants approached based on sampling protocol}

***^f^*** Number of participants classified based on their current zone of residence. This number varies from the ‘Number of participants responded (based on sampling zone)’, because the digital profiles were not updated about their current residence. There were participants who migrated from the sampling zone to their current residence’ zone.

***^g^*** Migration proportion among respondents = $\frac{\{Responses obtained as per zonal migration (based on current residence) -Number of participants responded \left( based on sampling zone \right)\}}{Number of participants responded \left( based on sampling zone \right)}$
