# Supplementary material for: Has loneliness and poor resilient coping influenced the magnitude of psychological distress among apparently healthy Indian adults during the lockdown? Evidence from a rapid online nation-wide cross-sectional survey
Source: PLoS One. 2021 Jan 14;16(1):e0245509. doi: 10.1371/journal.pone.0245509 (PMC7808626; doi:10.1371/journal.pone.0245509)
Supplement: S2 File — (DOCX) [file pone.0245509.s002.docx]

**Validation sample and result of test-retest reliability:**

**Table S2.1. Day-wise distribution of the validation sample depicting to number and response rate.**

| Study period (date) | Survey response | Validation sample | Validation Response | Response proportion in validation sample |
| --- | --- | --- | --- | --- |
| 4/17/2020 | 21 | 3 | 3 | 100.00% |
| 4/18/2020 | 59 | 6 | 5 | 83.33% |
| 4/19/2020 | 99 | 10 | 10 | 100.00% |
| 4/20/2020 | 119 | 20 | 19 | 95.00% |
| 4/21/2020 | 81 | 10 | 8 | 80.00% |
| 4/22/2020 | 103 | 12 | 11 | 91.67% |
| 4/23/2020 | 78 | 10 | 8 | 80.00% |
| 4/24/2020 | 47 | 5 | 5 | 100.00% |
| 4/25/2020 | 67 | 9 | 7 | 77.78% |
| 4/26/2020 | 76 | 11 | 7 | 63.64% |
| 4/27/2020 | 91 | 15 | 10 | 66.67% |
| 4/28/2020 | 88 | 9 | 9 | 100.00% |
| 4/29/2020 | 48 | 6 | 6 | 100.00% |
| 4/30/2020 | 31 | 4 | 3 | 75.00% |
| 5/1/2020 | 19 | 2 | 2 | 100.00% |
| 5/2/2020 | 12 | 2 | 2 | 100.00% |
| 5/3/2020 | 19 | 2 | 2 | 100.00% |
| 5/4/2020 | 35 | 5 | 4 | 80.00% |
| 5/5/2020 | 51 | 5 | 5 | 100.00% |
| 5/6/2020 | 24 | 3 | 2 | 66.67% |
| 5/7/2020 | 18 | 2 | 2 | 100.00% |
| 5/8/2020 | 9 | 1 | 1 | 100.00% |
| 5/9/2020 | 17 | 2 | 2 | 100.00% |
| 5/10/2020 | 14 | 2 | 2 | 100.00% |
| 5/11/2020 | 12 | 2 | 2 | 100.00% |
| 5/12/2020 | 7 | 1 | 1 | 100.00% |
| 5/13/2020 | 4 | 1 | 1 | 100.00% |
| 5/14/2020 | 0 | 0 | 0 | -- |
| 5/15/2020 | 0 | 0 | 0 | -- |
| 5/16/2020 | 0 | 0 | 0 | -- |
| Overall | 1249 | 160 | 139 | 86.88% |

**Table S2.2. Reliability (test of agreement) of the study questions based on test-retest among the validation sample.**

| Domain | Items in the questionnaire | Item characteristic | Kappa coefficient | Intra-Class Correlation coefficient |
| --- | --- | --- | --- | --- |
|  | **Identification of zone of residence based on current address of the participant** | *Categorical* | 0.95 |  |
| Background information | **Age** | *Continuous* |  | 0.91 |
|  | **Gender** | *Categorical* | 0.99 |  |
|  | **Area of residence** | *Categorical – binary* | 0.99 |  |
|  | **Marital status** | *Categorical* | 0.97 |  |
|  | **Educational status** | *Categorical* | 0.91 |  |
|  | **Living arrangement** | *Categorical* | 0.87* |  |
|  | **Occupation (Employment)** | *Categorical* | 0.9 |  |
|  | **Going to workplace or institution regularly** | *Categorical – binary* | 0.99 |  |
| Information on COVID19 | **Sources of information regarding COVID19** | *Categorical* | 0.88* |  |
|  | **Any confirmed and/or suspect case of COVID19 in the immediate surrounding and/or family** | *Categorical* | 0.84* |  |
| Perceptions | **Perception about effect (severity) of pandemic in terms of severity** | *Ordinal (considered continuous as data distributed on 5-point Likert-type scale)* |  | 0.9 |
|  | **Perception about effectiveness of lockdown in controlling the pandemic** | *Ordinal (considered continuous as data distributed on 5-point Likert-type scale)* |  | 0.92 |
|  | **Perception about impact (seriousness) of implementation of physical distancing measures** | *Ordinal (considered continuous as data distributed on 5-point Likert-type scale)* |  | 0.95 |
| Psychological distress questionnaire | **About how often did you feel Nervous?** | *Ordinal (considered continuous as data distributed on 5-point Likert-type scale)* |  | 0.94 |
|  | **About how often did you feel hopeless?** | *Ordinal (considered continuous as data distributed on 5-point Likert-type scale)* |  | 0.96 |
|  | **About how often did you feel restless or fidgety?** | *Ordinal (considered continuous as data distributed on 5-point Likert-type scale)* |  | 0.96 |
|  | **About how often did you feel so depressed that nothing could cheer you up?** | *Ordinal (considered continuous as data distributed on 5-point Likert-type scale)* |  | 0.96 |
|  | **About how often did you feel that everything was an effort?** | *Ordinal (considered continuous as data distributed on 5-point Likert-type scale)* |  | 0.93 |
|  | **About how often did you feel worthless?** | *Ordinal (considered continuous as data distributed on 5-point Likert-type scale)* |  | 0.97 |
|  | **Overall psychological distress** | *Categorical - binary* | Perfect agreement | |
| Brief resilient coping scale | **I look for creative ways to alter difficult situations.** | *Ordinal (considered continuous as data distributed on 5-point Likert-type scale)* |  | 0.86 |
|  | **Regardless of what happens to me, I believe I can control my reaction to it.** | *Ordinal (considered continuous as data distributed on 5-point Likert-type scale)* |  | 0.9 |
|  | **I believe I can grow in positive ways by dealing with difficult situations.** | *Ordinal (considered continuous as data distributed on 5-point Likert-type scale)* |  | 0.85 |
|  | **I actively look for ways to replace the losses I encounter in life.** | *Ordinal (considered continuous as data distributed on 5-point Likert-type scale)* |  | 0.95 |
|  | **Overall resilient coping category** | *Ordinal (three point ranked)* | Perfect agreement | |
| Loneliness scale | **How often do you feel that you lack companionship?** | *Ordinal (data distributed on 3-point Likert-type scale)* | Perfect agreement | |
|  | **How often do you feel left out?** | *Ordinal (data distributed on 3-point Likert-type scale)* |  | 0.91 |
|  | **How often do you feel isolated from others?** | *Ordinal (data distributed on 3-point Likert-type scale)* |  | 0.9 |
|  | **Overall loneliness category computed** | *Categorical - binary* | 0.97 |  |

* Mean agreement calculated for all the available options for multiple options items.
